# Dental Age and Tooth Development in Orthodontic Patients with Agenesis of Permanent Teeth

**DOI:** 10.1155/2017/8683970

**Published:** 2017-02-26

**Authors:** Jozo Badrov, Tomislav Lauc, Enita Nakaš, Ivan Galić

**Affiliations:** ^1^School of Medicine, University of Split, 21000 Split, Croatia; ^2^Study of Anthropology, Faculty of Social Sciences and Humanities, University of Zagreb, 10000 Zagreb, Croatia; ^3^Department of Dental Medicine, Faculty of Medicine, University of Osijek, 31000 Osijek, Croatia; ^4^Department of Orthodontics, School of Dental Medicine, University of Sarajevo, 71000 Sarajevo, Bosnia And Herzegovina

## Abstract

*Objective*. To compare the development of permanent teeth in a group of children with the congenitally missing permanent teeth (CMPT) and corresponding nonaffected group.* Methods*. The formation stages of all developing permanent teeth were determined on 345 panoramic radiographs (OPTs) by the method of Haavikko (1970), and dental age was calculated. The paired samples *t*-test was used to compare the differences between dental age (DA) and chronological age (CA) in those with CMPT and those not affected. Spearman test was used to evaluate the correlation between DA-CA and the number of missing teeth. The Wilcoxon signed rank test was used to compare the development of the teeth adjacent to the place of the agenesis with matched pair in corresponding nonaffected group.* Results*. Dental age was significantly delayed in CMPT children compared to the nonaffected group (*p* < 0.001). The mean differences were −0.57 ± 1.20 years and −0.61 ± 1.23 years in males and females, without difference between sexes (*p* = 0.763). The number of missing teeth affected the delay only in females (*p* = 0.024). Only mesial teeth in females were significantly delayed in development when compared to the nonaffected group (*p* = 0.007).* Conclusion*. Our findings show that the development of the permanent teeth is delayed when compared to the nonaffected group of the same sex and age.

## 1. Introduction

Congenitally missing permanent teeth (CMPT) or hypodontia is the most common anomaly of the permanent dentition [1, 2]. It is a failure of initial formation of tooth germ, causing permanent missing of the teeth. It could be associated with tens of different syndromes and craniofacial anomalies [3]. An etiology of familiar or nonsyndromic CMPT is not fully explained, and multifactorial inheritance including mutations of specific genes,* AXIN2*,* MSX1*,* PAX9*, and* WNT10A*, was reported [4–6]. The most common CMPT is nonsyndromic and affects a small number of teeth. A recent meta-analysis of the prevalence of CMPT demonstrated variability when comparing results for different continents, from 13.4% in Africa to 4.4% in Latin America and the Caribbean [7]. The most frequently CMPT are lower second premolars and upper lateral incisors, following upper second premolars and lower central incisors [7]. The development of permanent dentition, except third molars, can last up to 15 years of age, so it is important to recognize this pattern for timely treatment and particularly for the management of severe cases [8]. Dental methods for age calculation on developing teeth are important in the estimation of chronological age in cases of unknown date of birth, adoption of children, asylum seeking procedures, unaccompanied children, or estimating age from skeletal remains [9, 10]. Garn et al. [11] first reported a pattern of delayed dental development in children with CMPT. Some previous studies reported a significant delay of dentition development in children with CMPT when compared with their case-control pairs, while other showed no significant difference [12–17]. Odagami et al. [17] reported a significant association between severity of CMPT and delay of dental development while Uslenghi et al. [13], besides an association of the number of the missing teeth and dental delay, additionally showed a significant delay of both mesial and distal teeth adjacent to the missing tooth. Different age estimation methods were used to study dental development in children with CMPT, and most studies applied Haavikko staging system [15, 18].

Reported results of delay in dental development, from three months to two years, varied in sample size and cohort, staging system and statistical significance [15, 19]. A significant delay in dental development, especially in cases with severe CMPT, can provide valuable information for the beginning of orthodontic treatment. Tooth development in orthodontic patients with CMPT was not previously evaluated in Southern Croatia on a cross-sectional sample. The aims of this study were to examine the radiographic development of permanent teeth in orthodontic patients with CMPT, excluding third molars, to test the association of the number of the missing teeth to the dental development and how it affects the development of the teeth mesial and distal to the space of agenesis of the tooth.

## 2. Materials and Methods

This retrospective cross-sectional study was based on the evaluation of pretreatment orthopantomogram (OPT) of the orthodontic patients with CMPT. Digital OPTs were recorded during the period between 2008 and mid-2015 from six different orthodontic practices in Southern Croatia. The evaluated sample consisted of 4430 OPTs, while the sample with CMPT consisted of 345 OPTs of the children aged from 6 to 15 years, 149 males (6.5%) and 196 females (9.2%), Table 1. In total, 287 and 384 missing teeth were in 149 males and 196 females. Prevalence of 1 or 2 missing permanent teeth in evaluated sample was 66 (44.3%) and 56 (37.6%) in males and 83 (42.3%) and 75 (38.3%) in females. The mandibular teeth were significantly more affected than maxillary, 191 versus 96 and 246 versus 138 while left and right sides were similarly affected, 141 versus 146 and 200 versus 184 in males and females, respectively. The occurrence of bilateral CMPT of lower second premolars and upper second incisors was more common than unilateral CMPT.

The mean ages were 10.65 ± 2.15 years and 10.58 ± 2.03 in males and females, respectively (*p* = 0.780). For each participant with CMPT, OPT of the child not affected with CMPT of the same age and sex (control sample) was matched with the whole sample evaluated in this study. The detailed prevalence and teeth distribution of the children with CMPT in Southern Croatia will be separately published. The data for analysis of the sample included the date of birth and OPT, sex, the specific type, and a total number of missing permanent teeth in each participant with CMPT. We excluded all those with cleft lip and palate, congenital syndromes, and conditions related to CMPT from further analysis. A final sample consisted of 690 OPTs, half with CMPT and half not affected (Table 1).

The development of the permanent teeth in the final sample, except third molars, was evaluated by the Haavikko stages and median ages of the teeth from the upper and lower jaw [18]. Specifically, Haavikko [18] published a method, based on the evaluation of the development of six stages of the crown and six stages of the root and published median ages with 90% confidence intervals (CI) for each permanent tooth from upper and lower jaw. We used this data of age by Haavikko to calculate dental age as the mean age of all existing permanent teeth in the sample with CMPT while dental age not affected was calculated with an exclusion of those missing teeth in CMPT matching pair. All teeth with apex closure or stage “Ac” were excluded from the calculation of dental age. The difference between dental (DA) and chronological age (CA) or DA-CA was compared with paired samples *t*-test in both sexes. Additionally, the effect of severity of CMPT on DA-CA was evaluated by Spearman correlation coefficients. To investigate affection of the teeth adjacent to the missing ones, we analyzed OPTs with a single tooth missing in one quadrant and not more than missing two permanent teeth, excluding the first incisors and second molars [13]. For this purpose, we compared the stages of corresponding mesial and distal tooth of the place of agenesis to the same teeth of nonaffected participant. Wilcoxon singed-rank test was used to compare developmental stages. Kappa scores were used to examine intraobserver agreement of Haavikko stages on randomly selected 30 OPGs by the first author after four weeks without knowledge of age and sex.

## 3. Results

There was no difference between mean chronological age in CMPT and nonaffected groups for males (*p* = 0.603) and females (*p* = 0.393). Dental age, calculated by using the Haavikko standards, was underestimated in both CMPT and nonaffected samples.

**Figure 1:**
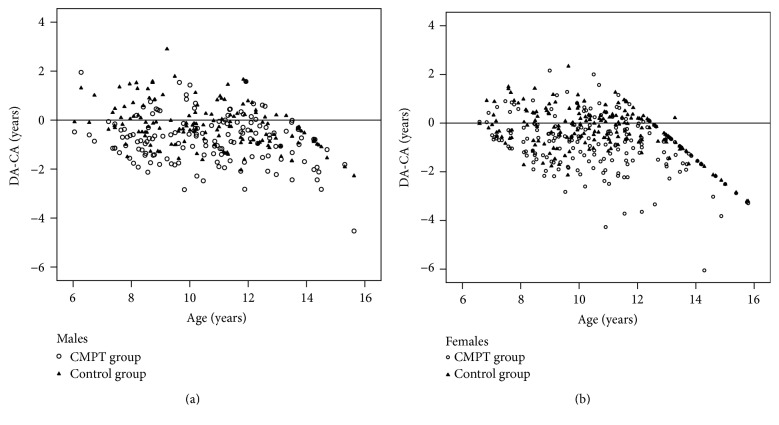
Scatterplot of difference between dental and chronological age (DA-CA) and chronological age (age) for the congenitally missing permanent teeth (CMPT) group and nonaffected group (control group).

Principally, dental age in both sexes was underestimated more in CMPT group, which is statistically significant, Table 2 and Figure 1. Dental development was more delayed in the CMPT children than in nonaffected CMPT (<0.001); the main difference was −0.57 ± 1.20 years and −0.61 ± 1.23 years in males and females without significant difference between sexes (*p* = 0.763).

**Figure 2:**
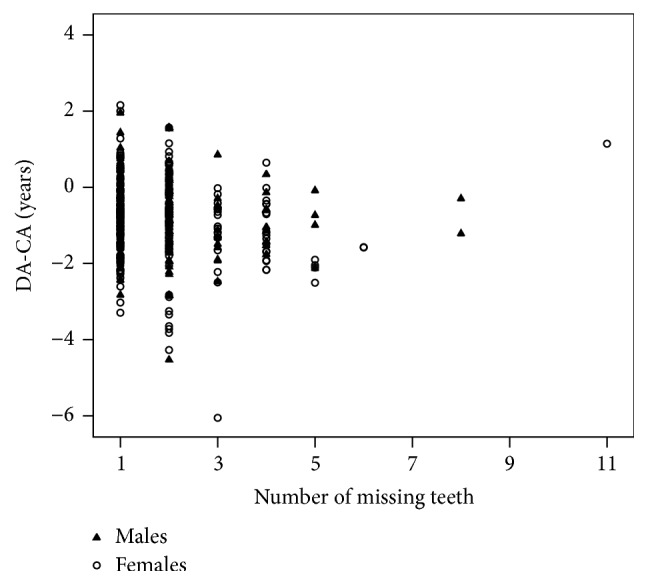
Scatterplot of difference between dental and chronological age (DA-CA) and a number of the congenitally missing permanent teeth in males and females.

Figure 2 shows the differences between dental and chronological age and a total number of missing teeth. The majority of the children have one or two missing teeth. The delay in dental development was significantly correlated with the severity of CMPT in females (*p* = 0.024) while in males was not significant (*p* = 0.451).

**Figure 3:**
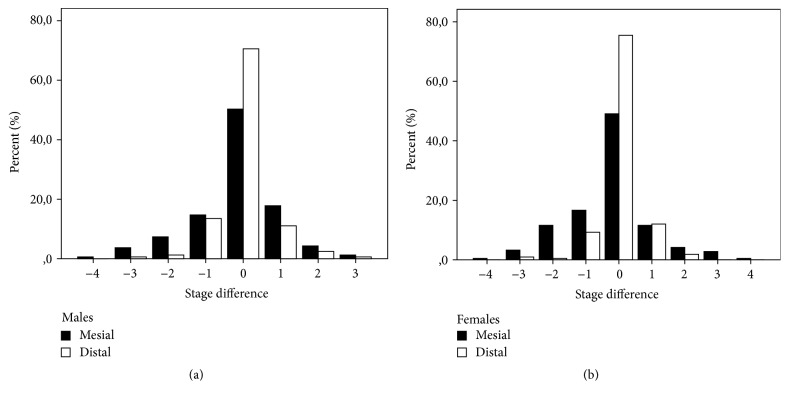
Distribution of stage differences of adjacent teeth in males and females.

Adjacent teeth to the place of missing showed a different pattern in sexes. In males, there were no significant delays in neither mesial (*Z* = −1.39, *p* = 0.166) nor distal teeth (*Z* = −0.28, *p* = 0.978). In females mesial teeth are significantly delayed (*Z* = −2.72, *p* = 0.007) while distally teeth were without significant difference (*Z* = −0.60, *p* = 0.547). The greatest difference was at one stage, up to four stages of delay, Figure 3.

The Kappa scores of intraobserver agreement varied between 0.51 for the tooth number 35 and 0.91 for the tooth number 36, with a mean value of 0.68 for maxillary and 0.70 for mandibular teeth which are substantial agreements according to Landis and Koch, Table 3 [20].

## 4. Discussion

We found a delay in dental development of −0.57 years and −0.61 years in the CMPT group when compared to nonaffected group in males and females, respectively. We also found the similar affection between the left and right sides of the jaws and greater affection of the lower permanent teeth versus the upper. Delayed dental development in orthodontic patients with CMPT may influence the beginning of clinical treatment, treatment plan, and the duration of therapy. Delayed dental age was also reported in males with constitutional delay of growth and puberty [21]. Kan et al. [19] hypothesized that dental delay in children with nonsyndromic CMPT indicates that CMPT may be an expression of disturbance of dental development. Clinical cases with severe hypodontia require both orthodontic correction and implant placement after ending of delayed dental and maxillofacial development [19]. It is still not clear what is the minimal clinically important and biologically relevant difference in a dental age that could affect orthodontic treatment plan and the results of dental age estimation in children with CMPT [22]. However, given the age range of observed children in this study, the difference in dental age of 0.6 years between the CMPT and control groups corresponds to 6% of the observed age range. A difference which is higher than 5% of a range size has been defined as the minimal clinically important differences in other clinical studies as well [23]. Age estimation method in living or dead based on an assessment of mineralization of permanent teeth may not be implemented in case of subjects with CMPT. Most of the methods use lower permanent teeth, and these are the teeth most likely to be affected with agenesis.

The delay was smaller than that in other studies which used the Haavikko method. Uslenghi et al. [13] reported the delay of −1.53 years for the total sample and Rune and Sarnäs [12] reported −1.8 years for males and −2.0 years for females. Ruiz-Mealin et al. [24] used Haavikko and Demirjian stages and reported also underestimated dental age when compared to nonaffected group. Principally, dental age was underestimated by −0.88 years in males and −0.60 years in females for Haavikko method and by −0.84 years for males and −0.87 years for females for Demirjian method [24]. Tunç et al. [25] applied Demirjian standards and also found the delay in the children with hypodontia when compared to nonaffected group; the mean delay did not exceed 0.3 years in either sex. Odagami et al. [17], in their study on 77 males and 100 females using Moorrees radiographic stages, also showed the delay of dental development which was not statistically significant. Lozada Riascos and Infante Contreras [16] also reported the insignificant delay in dental development of 0.7 years for males and 1.0 years for females. The most recent Danish study also reported −0.37 to −0.50 years in dental development when compared to nonaffected dentition [14].

A significant association between severity of agenesis and delay in development was found in females in our study while Uslenghi et al. [13] also reported a significant association, without an evidence of the difference for the specific sex. Odagami et al. [17] also reported a significant association, while the study of Lozada Riascos and Infante Contreras [16] found no significant association between development and sex or the number of missing teeth. Tunç et al. [25] found no correlation between the differences in dental and chronologic age and the severity of CMPT.

Our study showed a different pattern in delay of the teeth adjacent to the place of the agenesis. Only females demonstrated a statistically significant delay in the distal adjacent teeth. Uslenghi et al. [13] revealed that the teeth adjacent to the position of the agenesis, both mesial and distal, were significantly delayed compared to the corresponding teeth in the matched group. Daugaard et al. [26] evaluated dental maturity in the mandibular canine, premolar, and molar innervation fields in children with agenesis of the mandibular second premolars, by using Haavikko's approach. A development of canines was delayed in those with unilateral CMPT, with a larger delay in females, while the second molar was not delayed in males but was in females [26].

In orthodontic practice, it is important to understand normal dental development clearly and to recognize those patients with agenesis to plan orthodontic treatment in a proper manner, with right starting time and duration [15]. Population studies have investigated a large number of children who were orthodontic patients. Although the proportion of those with hypodontia was higher compared to the general population, data collected on this orthodontic population is considered to be reliable and was included in the meta-analysis of the prevalence of CMPT [7]. Evidence of difference in the dental development of the children with CMPT should be taken into account when calculating the dental age for different purposes because various dental methods have been recognized as a reliable approach for estimating biological maturity. Dental age can help estimate someone's age in forensic, civil, and archaeological investigations.

## 5. Conclusion

A delay of −0.57 years in females and −0.61 years in males in dental age was found in the children with CMPT compared to nonaffected group (*p* < 0.001). A delay was noticed in females in the mesial teeth adjacent to the location of CMPT compared to the teeth from the control group (*p* = 0.024). Only females showed a significant correlation between the number of missing teeth and severity of delay of development (*p* = 0.007). These findings should be taken into account because they can impact the orthodontic treatment plan and the results of dental age estimation in children with CMPT.

## Figures and Tables

**Table 1:** Distribution of participants with congenitally missing permanent teeth (CMPT), control sample of nonaffected children (control), and evaluated sample across different age groups.

Age group (years)	Males				Females				Total			
	*N*_CMPT_	*N*	%	*N*_CONTROL_	*N*_CMPT_	*N*	%	*N*_CONTROL_	*N*_CMPT_	*N*	%	*N*_CONTROL_
6.0–6.9	4	46	8.7	4	3	54	5.6	3	7	100	7.0	7
7.0–7.9	13	168	7.7	13	18	72	25.0	18	31	240	12.9	31
8.0–8.9	27	150	18.0	27	26	149	17.4	26	53	299	17.7	53
9.0–9.9	15	315	4.8	15	32	167	19.2	32	47	482	9.8	47
10.0–10.9	21	390	5.4	21	35	328	10.7	35	56	718	7.8	56
11.0–11.9	26	286	9.1	26	33	408	8.1	35	59	694	8.5	59
12.0–12.0	21	200	10.5	21	22	400	5.5	22	43	600	7.2	43
13.0–13.9	12	588	2.0	12	16	268	6.0	16	28	856	3.3	28
14.0–14.0	8	117	6.8	8	7	216	3.2	7	15	333	4.5	15
15.0–15.9	2	40	5.0	2	4	68	5.9	4	6	108	5.6	6

Total	149	2300	6.5	149	196	2130	9.2	196	345	4430	7.8	345

*N*_CMPT_, a number of participants with CMPT; *N*_CONTROL_, a number of nonaffected participants; *N*, a total number of participants.

**Table 2:** Underestimation of dental and chronological age (DA-CA) in the children with congenitally missing permanent teeth (CMPT) and nonaffected children (control).

Sex	*N*	CA (years)	DA (years)	DA-CA (years)	*t* (df)	*p*
Males_CMPT_	149	10.65 ± 2.15	9.85 ± 2.17	−0.80 ± 0.97	−10.11 (148)	<0.001
Males_CONTROL_		10.65 ± 2.15	10.42 ± 1.96	−0.23 ± 0.90	−3.10 (148)	0.002

Females_CMPT_	196	10.59 ± 2.04	9.70 ± 1.92	−0.88 ± 1.14	−10.81 (195)	<0.001
Females_CONTROL_		10.58 ± 2.03	10.31 ± 1.83	−0.27 ± 0.89	−4.25 (195)	<0.001

Total_CMPT_	345	10.61 ± 2.08	9.77 ± 2.02	−0.85 ± 1.07	−14.71 (344)	<0.001
Total_CONTROL_		10.61 ± 2.08	10.36 ± 1.88	−0.25 ± 0.89	−5.24 (344)	<0.001

*N*, a number of participants; CA, chronological age; DA, dental age; DA-CA, difference between DA and CA; *t*, paired samples *t*-test; df, degrees of freedom.

**Table 3:** Kappa scores for intraobserver agreement of the evaluated teeth on the randomly selected 30 orthopantomograms.

*Maxillary teeth*	*17*	*16*	*15*	*14*	*13*	*12*	*11*	*21*	*22*	*23*	*24*	*25*	*26*	*27*
Kappa score	0.68	0.76	0.65	0.74	0.68	0.55	0.76	0.81	0.61	0.68	0.79	0.56	0.71	0.57

*Mandibular teeth*	*47*	*46*	*45*	*44*	*43*	*42*	*41*	*31*	*32*	*33*	*34*	*35*	*36*	*37*
Kappa score	0.72	0.81	0.61	0.77	0.68	0.75	0.66	0.65	0.69	0.76	0.73	0.51	0.91	0.60
